# Outcomes and factors associated with mortality in patients with atrial fibrillation and heart failure: FARAONIC study

**DOI:** 10.1002/clc.24106

**Published:** 2023-08-18

**Authors:** Juan José Gómez Doblas, José María Cepeda‐Rodrigo, Rosa Agra Bermejo, Elvira Blanco Labrador, Maria Teresa Blasco, Margarita Carrera Izquierdo, Iñaki Lekuona, Alejandro Recio Mayoral, Carles Rafols, Nicolás Manito

**Affiliations:** ^1^ Cardiology Department Hospital Clínico Universitario Virgen de la Victoria Málaga Spain; ^2^ Centro de Investigación Biomédica en Red de Enfermedades Cardiovasculares CIBERCV Madrid Spain; ^3^ Department of Internal Medicine Hospital Vega Baja Orihuela Spain; ^4^ Cardiology Department Hospital Universitario de Santiago de Compostela A Coruña Spain; ^5^ Cardiology Department Complexo Hospitalario de Ourense Ourense Spain; ^6^ Cardiology Department Hospital Universitario Miguel Servet Zaragoza Spain; ^7^ Department of Internal Medicine Complejo Asistencial Universitario de Soria Soria Spain; ^8^ Cardiology Department Hospital Galdakao‐Usansolo Bizkaia Spain; ^9^ Cardiology Department Hospital Universitario Virgen Macarena Sevilla Spain; ^10^ Medical Department Bayer Hispania Barcelona Spain; ^11^ Cardiology Department Hospital Universitario de Bellvitge Barcelona Spain

**Keywords:** anticoagulation, atrial fibrillation, death, direct oral anticoagulant, heart failure, hemorrhage, rivaroxaban, thromboembolism, worsening heart failure

## Abstract

**Background:**

Heart failure (HF) and atrial fibrillation (AF) are common and coexistent conditions.

**Hypothesis:**

To investigate the adverse events and mortality risk factors in patients with AF and HF treated with rivaroxaban in Spain.

**Methods:**

Multicenter, prospective and observational study with a follow‐up of 2 years, that included adults, with a diagnosis of nonvalvular AF and chronic HF, anticoagulated with rivaroxaban at least 4 months before being enrolled.

**Results:**

A total of 672 patients from 71 Spanish centers were recruited, of whom 658 (97.9%) were included in the safety analysis and 552 (82.1%) in the per protocol analysis. At baseline, the mean age was 73.7 ± 10.9 years, 65.9% were male, 51.3% had HF with preserved ejection fraction and 58.7% were on New York Heart Association functional class II. CHA_2_DS_2_‐VASc was 4.1 ± 1.5. During the follow‐up, 11.6% of patients died and around one‐quarter of patients were hospitalized or visited the emergency department, being HF worsening/progression the main cause (51.1%), with a 2.9% of thromboembolic events and 2.0% of acute coronary syndromes. Major bleeding occurred in 3.1% of patients, with 0.5% experiencing intracranial bleeding but no fatalities. Compliance with HF treatment was associated with a lower risk of death (hazard ratio: 0.092; 95% confidence interval: 0.03–0.31).

**Conclusions:**

Among patients with HF and AF anticoagulated with rivaroxaban, incidences of thromboembolic or hemorrhagic complications were low. The most important factor for improving survival was compliance with HF drugs, what strengths the need for early treatment with HF disease‐modifying therapy and anticoagulation.

## Graphical abstract

Multicenter, prospective and observational study with a follow‐up of 2 years that included 672 adults with a diagnosis of atrial fibrillation (AF) and chronic heart failure (HF), anticoagulated with rivaroxaban. At baseline, the mean age was 73.7 ± 10.9 years, 65.9% were male, 51.3% had HF with preserved ejection fraction and 58.7% were on New York Heart Association functional class II. CHA_2_DS_2_‐VASc was 4.1 ± 1.5. During the follow‐up, 24.9% of patients were hospitalized or visited the emergency department for HF, 11.6% died, 2.9% had a thromboembolic event, 1.1% a stroke, 3.1% a major bleeding, 0.5% an intracranial bleeding and no fatal bleeding occurred. Compliance with HF treatment was associated with a lower risk of death (hazard ratio: 0.092; 95% confidence interval: 0.03–0.31).

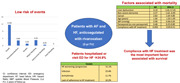

## 1 INTRODUCTION

Heart failure (HF) and atrial fibrillation (AF) are two common conditions that frequently coexist.^1^, ^2^ In fact, it has been estimated that up to 30% of patients with AF have HF, and conversely, around one‐third of patients with HF have concomitantly AF.^3^, ^4^, ^5^, ^6^ In fact, both entities share many risk factors (i.e., hypertension, diabetes, aging, obesity, etc.) and exhibit interrelated mechanisms and pathophysiology. Thus, HF increases the risk of developing AF through the elevation of left atrial pressure, promoting conduction abnormalities and fibrosis on the contrary, AF leads to a reduction in cardiac output secondarily to different mechanisms, including loss of atrial contraction, rapid ventricular rate, irregular ventricular filling, and tachycardia‐induced cardiomyopathy.^1^, ^2^

Patients with AF and HF have an increased risk of stroke, hospitalization for HF exacerbations, and all‐cause mortality.^7^, ^8^ To reduce the thromboembolic risk in this population, guidelines recommend oral chronic anticoagulation.^9^ Unfortunately, despite anticoagulation, the risk of adverse cardiovascular events remains high.^7^, ^8^ However, the majority of this information provides from studies in which patients were anticoagulated with vitamin K antagonists.^8^ As a result, it is uncertain whether these figures can be applied to patients taking direct oral anticoagulants.

The ROCKET‐AF trial showed that among very high stroke‐risk patients, rivaroxaban was effective and safe compared with warfarin.^10^ In a specific analysis of the ROCKET‐AF trial performed according to HF status, the relative efficacy and safety of rivaroxaban versus warfarin were independent of the presence of previous HF.^11^ In clinical practice, a retrospective database study with rivaroxaban in patients with AF showed that rivaroxaban significantly reduced the risk of HF hospitalization and overall mortality compared with vitamin K antagonists.^12^ However, prospective data regarding outcomes in patients with HF and AF taking rivaroxaban in real‐life patients are lacking.

The aim of this study was to investigate the incidence of adverse events (all‐cause mortality and hospitalizations, acute decompensated HF (emergency department visits and hospitalizations), thromboembolic events, acute coronary syndrome, and hemorrhages), as well as to determine mortality risk factors (all‐cause death) in patients with AF and HF treated with rivaroxaban in Spain.

## 2 METHODS

### 2.1 Design of the study and study population

Multicenter, prospective, observational, noninterventional, and cohort study, developed in 71 centers from Spain. Patients that met with the inclusion/exclusion criteria were consecutively recruited during a routine follow‐up visit between March 2018 and July 2019. Patients were followed‐up during 2 years (baseline, follow‐up visits 1‐to‐3, and end of study), according to routine practice. Adults with nonvalvular AF and chronic HF (regardless of New York Heart Association [NYHA] functional class or ejection fraction) that received rivaroxaban for stroke prevention, at least 4 months before being enrolled in the study and that gave written informed consent, were included. The exclusion criteria were patients participating in a clinical trial, who started treatment with rivaroxaban after the start of the inclusion period or within the last 4 months before inclusion, with significant mitral stenosis or mechanical prosthesis, or with severe cognitive impairment. The study was approved by the research ethical committee of Parc de Salut Mar, on November 14, 2017.

### 2.2 Baseline variables

Data were collected from the electronic clinical history of patients, or during the interview in the routine visit and recorded into a specific electronic case report form. Biodemographic data (age, gender), AF data (time since AF diagnosis, type of AF, CHA_2_DS_2_‐VASc score,^13^ HAS‐BLED score^14^), HF data (time since HF diagnosis, NYHA functional class, type of HF—reduced, mildly reduced or preserved ejection fraction—^15^), cardiovascular risk factors (arterial hypertension, hyperlipidemia, diabetes mellitus, smoking), other comorbidities (previous coronary artery disease, cerebrovascular disease, chronic kidney disease), HF treatments (diuretics, renin‐angiotensin system inhibitors, beta‐blockers, mineralocorticoid receptor antagonists, digoxin, ivabradine), as well as the information regarding treatment with rivaroxaban along the study, including the dose and medication persistence were recorded.

### 2.3 Outcomes

The proportion of patients that were hospitalized or visited the emergency department (HF and non‐HF‐related), during the follow‐up, the mean number of hospitalizations/visits among those patients with an event, as well as the causes of hospitalization/visits to the emergency department, were recorded. In addition, the proportion of patients that died during the study or that had a thromboembolic event (arterial or venous thrombosis), an acute coronary syndrome, or a hemorrhagic event (major bleeding,^16^ intracranial bleeding, or fatal hemorrhage) were also determined. The factors potentially influencing the risk of death were analyzed and included all baseline data.

### 2.4 Statistical analysis

Three types of analysis populations were defined in this study: (1) Safety analysis set: all patients that had received rivaroxaban ≥4 months before being enrolled in the study; (2) full analysis set: all patients that had received rivaroxaban ≥4 months before being enrolled in the study and who had satisfied the inclusion/exclusion criteria. This population was used for the analysis of the main objective of the study; (3) per protocol set: all patients that had received rivaroxaban ≥4 months before being enrolled in the study, who had satisfied the inclusion/exclusion criteria and that had had ≥1 postbaseline visit, except for premature terminations due to death or adverse events. This population was used for the baseline description and the analyses of the main objectives of the study.

The qualitative variables were defined by their absolute and relative frequencies and the quantitative variables by measures of central tendency (mean or median) and dispersion (standard deviation or interquartile range), as required. To explore the factors associated with mortality, baseline variables, including demography, vital signs, comorbidities, and concomitant treatments, were considered for inclusion in a Cox proportional hazard model. The Cox model was computed by considering mortality after the baseline visit. Initially, the feasibility of the factors was explored using bivariate models. Then, those variables with a signification level lower than 0.15 were included in the multivariate models. Only the significant factors (*p* < .05) were finally considered to build the models. All analyses are performed with SAS® version 9.4 (SAS Institute, Inc.).

## 3 RESULTS

**Figure 1:**
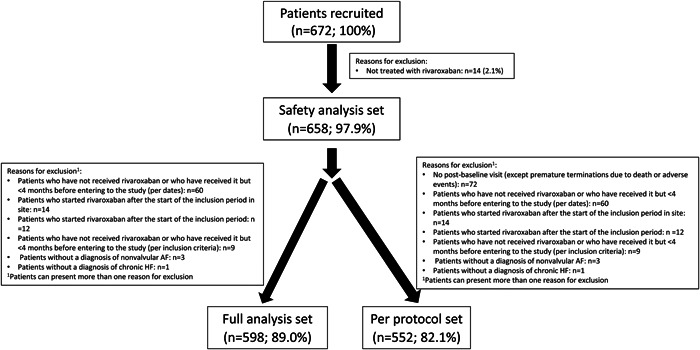
Flow chart of the study.

A total of 672 patients were recruited, of whom 658 (97.9%) patients were included in the safety analysis set, 598 (89.0%) in the full analysis set, and 552 (82.1%) in the per protocol set (Figure 1).

Baseline clinical characteristics are shown in Table 1. Mean age was 73.7 ± 10.9 years, 65.9% of patients were male, 53.9% had permanent AF, and 31.1% paroxysmal AF. Mean CHA_2_DS_2_‐VASc and HAS‐BLED scores were 4.1 ± 1.5 and 1.6 ± 0.9, respectively. The majority of patients were on NYHA functional class II (58.7%) and approximately half of the patients had HF with preserved ejection fraction. Regarding HF treatments, 85.5% were taking a renin‐angiotensin system inhibitor, mainly angiotensin‐converting enzyme inhibitors, 79.7% beta‐blockers, and 51.4% mineralocorticoid receptor antagonists. 69% of patients were taking rivaroxaban 20 mg and 31% rivaroxaban 15 mg. Only 6.9% had permanently discontinued treatment with rivaroxaban at the end of the follow‐up.

**Table 1:** Baseline clinical characteristics of the study population, per protocol set (*n* = 552).

Biodemographic data	
Age (years)	73.7 ± 10.9
Gender (male, %)	65.9
**AF data**	
Time since AF diagnosis (months), median (IQR)	39 (16.5–82.2)
Type of AF (%)	
Paroxysmal	31.1
Persistent	11.9
Long‐standing persistent	3.1
Permanent	53.9
CHA_2_DS_2_‐VASc score	4.1 ± 1.5
HAS‐BLED score	1.6 ± 0.9
**HF data**	
Time since HF diagnosis(months), median (IQR)	28 (11.8–67.1)
NYHA functional class, %	
Class I	17.4
Class II	58.7
Class III	23.2
Class IV	0.7
HF classification (%)	
HF with reduced ejection fraction	31.3
HF with mildly reduced ejection fraction	17.4
HF with preserved ejection fraction	51.3
**Cardiovascular risk factors**	
Arterial hypertension (%)	77.5
Hyperlipidemia (%)	54.7
Diabetes mellitus (%)	37.3
Current smoker and former ex‐smoker (<1 year) (%)	7.4
**Other comorbidities**	
Previous coronary artery disease (%)	39.1
Chronic kidney disease (%)	32.4
Previous cerebrovascular disease (%)	12.5
**HF treatments**	
Diuretics (%)	90.6
RAAS inhibitors (%)	85.5
Angiotensin‐converting enzyme inhibitors	36.7
Sacubitril/valsartan	25.0
Angiotensin II receptor blockers	23.8
Beta‐blockers (%)	79.7
Mineralocorticoid receptor antagonists (%)	51.4
Digoxin (%)	23.0
Ivabradine (%)	3.1
**Treatment with rivaroxaban**	
Time from start of treatment to study entering, months	25.5 ± 18.8
Dose (%)	
15 mg	31.0
20 mg	69.0
Permanent discontinuation (%)	6.9

Abbreviations: AF, atrial fibrillation; HF, heart failure; IQR, interquartile range; NYHA, New York Heart Association; RAAS, renin‐angiotensin system inhibitors.

Hospitalizations and/or visits to the emergency department during the follow‐up are presented in Table 2. Around one‐quarter of patients were hospitalized or visited the emergency department due to HF, being HF worsening/progression the main cause (51.1%). Half of the patients hospitalized/visited the emergency department due to non‐HF causes. With regard to outcomes, after 2 years of follow‐up, 11.6% of patients died, 2.9% had a thromboembolic event, 2.0% an acute coronary syndrome, 3.1% a major bleeding, 0.5% an intracranial bleeding and no patient died due to bleeding (Table 3). Liver dysfunction, nonsevere dementia, cancer, and increasing age were associated with mortality, whereas systolic blood pressure, paroxysmal AF (vs. nonparoxysmal), and mostly compliance with HF treatment (hazard ratio: 0.092; 95% confidence interval: 0.03–0.31) with a lower risk of death (Table 4).

**Table 2:** Hospitalizations and/or visits to the emergency department during the follow‐up.

*HF‐related*	
Patients that have hospitalized or visited the emergency department (%)	24.9
Mean number among those patients who hospitalized or visited the emergency department	1.8 ± 1.3
Causes (%)^a^	
HF worsening/progression	51.1
Infection	23.4
Arrhythmias	11.7
Lack of adherence to HF treatment	10.2
Uncontrolled hypertension	5.1
Acute coronary syndrome	4.4
Others	16.1
*Non‐HF related*	
Patients that have hospitalized or visited the emergency department (%)	49.7
Mean number among those patients who hospitalized or visited the emergency department	2.5 ± 2.6
Causes (%)^a^	
Noninfectious and infectious respiratory causes	27.7
Cardiovascular	20.9
Trauma/fall	16.4
Hemorrhages	13.9
Scheduled surgery	12.4
Nonrespiratory infections	11.3
Cancer	3.3
Others	69.7

Abbreviation: HF, heart failure.

^a^ Patients may present more than one reason.

**Table 3:** Events after 2 years of follow‐up.

Death (%)	11.6
Thromboembolic event (%)	2.9
Stroke	1.1
Transient ischemic attack	1.1
Systemic embolism	0.4
Deep venous thrombosis	0.2
Pulmonary embolism	0.2
Acute coronary syndrome (%)	2.0
Hemorrhagic event (%)	11.9
Major bleeding	3.1
Intracranial bleeding	0.5
Fatal hemorrhage	0

**Table 4:** Factors associated with mortality.

	HR	95% CI	*p* Value
Liver dysfunction	4.19	1.01–17.45	.049
Nonsevere dementia	3.37	1.62–6.99	.001
Cancer	2.83	1.64–4.89	.0002
Age (years), per each unit of the variable	1.06	1.03–1.09	<.0001
SBP (mm Hg), per each unit of the variable	0.98	0.96–0.99	.0009
Paroxysmal (vs. nonparoxysmal)	0.48	0.25–0.89	.021
Compliance with HF treatment	0.092	0.03–0.31	.0001

Abbreviations: CI, confidence interval; HR, hazard ratio; SBP, systolic blood pressure.

## 4 DISCUSSION

Our study showed in a wide sample of patients with AF and HF treated with rivaroxaban in Spain that the incidences of thromboembolism and bleeding were low, around one‐quarter of patients were hospitalized or visited the emergency department due to HF and approximately 1 out of 10 patients died in 2 years. Liver dysfunction, nonsevere dementia, cancer, and increasing age were predictors of death, whereas paroxysmal AF (low AF burden) and mainly compliance with HF treatment were associated with a lower risk of death.

In our study, more than 550 patients with AF and HF were finally analyzed. Patients were old (mean age 74 years), had a high thromboembolic risk (CHA_2_DS_2_‐VASc 4.1) and around half of the patients had HF with preserved ejection fraction and one‐third HF with reduced ejection fraction. This clinical profile is in line with that of those patients with HF included in ROCKET‐AF trial.^11^ Similarly, in real‐life patients, such as those patients with HF included in the global registry on long‐term oral antithrombotic treatment in patients with atrial fibrillation (GLORIA‐AF) registry (one‐quarter of the study population), patients were old, had a high thromboembolic risk and nearly 40% had HF with reduced ejection fraction.^17^ This was also in line with the subgroup of patients with HF included in the EMIR study, a Spanish registry of patients with AF treated with rivaroxaban in clinical practice.^18^ As a result, our data are representative of anticoagulated patients with HF and AF. On the other hand, previous studies have shown that within the overall HF population, the proportion of patients with HF with reduced ejection fraction is higher than that observed in our study. This could be related with the fact that both conditions, AF and HF with preserved ejection fraction are age‐related, and our patients were older.^19^, ^20^

With regard to HF treatments, the majority of patients were taking the appropriate disease‐modifying treatment, as guidelines recommend (86% a renin‐angiotensin system inhibitor, 80% a beta blocker, and half of patients a mineralocorticoid receptor antagonists).^15^ These numbers are higher than those reported in previous studies performed in the overall HF population.^5^, ^6^ Despite that, the number of hospitalizations and/or visits to the emergency department due to HF during the follow‐up persisted high, being HF worsening/progression the main cause. As a result, optimization of HF remains mandatory. In this context, the addition of SGLT2 inhibitors to recommended therapy has been associated with a reduction in the risk of worsening HF or cardiovascular death, independently of AF status.^21^, ^22^ More recently, vericiguat has been shown to be particularly effective in patients with worsening HF with reduced ejection fraction, regardless of the history of AF.^23^ Of note, as patients with AF and HF have an increased risk of events (vs. no AF patients), the absolute number of prevented events with these therapies may be greater in this population.^21^, ^22^, ^23^

On the other hand, half of patients hospitalized/visited the emergency department due to non‐HF causes (i.e., noninfectious and infectious respiratory causes, other cardiovascular events, trauma/fall, hemorrhages). Therefore, a more holistic approach is mandatory in patients with HF and AF to reduce morbidity.^9^ In this context, in our study rivaroxaban was associated with a low risk of bleeding after a 2‐year period, with only 3% of major bleeding, 0.5% of intracranial bleeding, and no patient with fatal hemorrhage. In the subgroup of HF patients taking rivaroxaban in the ROCKET‐AF trial, the rate of intracranial hemorrhage was 0.40 events per 100 patient years.^11^ In the EMIR study, a real‐world prospective registry of AF patients taking rivaroxaban in Spain, among patients with HF, annual rate of major bleeding was 1.4%.^18^ Remarkably, a study performed in AF patients at high risk for falls, treatment with rivaroxaban was associated with a marked reduction of intracranial hemorrhage compared to warfarin.^24^ In summary, all these data indicate that rivaroxaban can be safely used in patients with AF and HF.

With regard to outcomes, after 2 years of follow‐up, nearly 3% had a thromboembolic event (arterial or venous) and 2.0% an acute coronary syndrome. In the subgroup of HF patients taking rivaroxaban in the ROCKET‐AF trial, the rate of stroke or systemic embolization was 1.9 events per 100 patient‐years and the rate of myocardial infarction was 1.1 events per 100 patient‐years.^11^ Among those patients with HF included in the EMIR study, the annual rates of thromboembolic events (stroke + systemic embolism + transient ischemic attack) and major cardiovascular events were 1.2% and 3.0%, respectively.^18^ All these data clearly suggest that although AF patients with HF (vs. no HF) have a higher risk of adverse events,^11^, ^18^ incidences of thromboembolic complications and myocardial infarction are very low among patients treated with rivaroxaban. In fact, previous studies have suggested that compared to warfarin, rivaroxaban could provide further benefits reducing the risk of ischemic cardiac events in patients with AF.^25^ This could provide an added value in the comprehensive management of patients with AF and HF.

In our study, nearly 12% of patients had died at the end of the follow‐up. In the subgroup of HF patients taking rivaroxaban in the ROCKET‐AF trial, the rate of all‐cause death was 5.1 events per 100 patient‐years^11^ and in the HF population of the EMIR study, the annual rate of death was 5.5%.^18^ As a result, our data were consistent with previous studies. Considering that in our study, only 3% had a thromboembolic event, this means that the majority of deaths in patients with AF and HF chronically anticoagulated are nonstroke dependent and other conditions should be considering. Previous studies have shown that in HF patients, age, renal function, severity of HF, no prescription of HF drugs, diabetes, or lower systolic blood pressure, have been associated with a higher mortality risk.^26^, ^27^, ^28^ In our study, the most important factor associated with better survival was compliance with HF treatment. European guidelines undoubtedly recommend the use of HF disease‐modifying treatment, including, renin‐angiotensin system inhibitors, beta‐blockers, mineralocorticoid receptor antagonists, and SGLT2 inhibitors, as soon as possible to reduce morbidity and mortality, but also anticoagulation in AF patients, preferably with direct oral anticoagulants.^15^ Importantly, in our study medication persistence with rivaroxaban was high. In light of all these data, oral anticoagulation is mandatory and rivaroxaban seems safe in this population.

## 5 LIMITATIONS

As there is no comparator group, direct comparisons with other drugs cannot be achieved and only indirect hypothesis can be suggested. However, the objectives of the study can be adequately addressed with the current design of the study. On the other hand, the results of this study are applicable to patients anticoagulated with rivaroxaban, but not with other direct oral anticoagulants. Finally, as patients included in this study were representative of the Spanish population with HF and AF, the results can only be extended to patients with a similar clinical profile.

## 6 CONCLUSIONS

After 2 years of follow‐up, rates of thromboembolic or hemorrhagic complications were low, and approximately 1 out of 10 patients with HF and AF died. The most important factor for improving survival was compliance with HF treatment, what strengths the need for early treatment with HF disease‐modifying therapy, including anticoagulation. Rivaroxaban has been shown to be safe in this high‐risk population.

## AUTHOR CONTRIBUTIONS

All authors have contributed significantly to the work presented in this article, contributing to the conception, design, or acquisition of information, or to the analysis and interpretation of data. All the authors have participated in the drafting and/or revision of the manuscript and accept its publication.

## CONFLICT OF INTEREST STATEMENT

The authors received honoraria from Bayer Hispania SL for their participation as researchers in the FARAONIC study.

## Data Availability

The data that support the findings of this study are available from the corresponding author upon reasonable request.
